# Supplementary material for: The causal role of circulating inflammatory markers in osteoporosis: a bidirectional Mendelian randomized study
Source: Front Immunol. 2024 Jul 18;15:1412298. doi: 10.3389/fimmu.2024.1412298 (PMC11291241; doi:10.3389/fimmu.2024.1412298)
Supplement: Supplementary file 1 [file DataSheet_1.zip › supplementary material/OSTEOPOROSIS-CIM/GCST90274807.tsv.gz_leave_one_out.pdf]

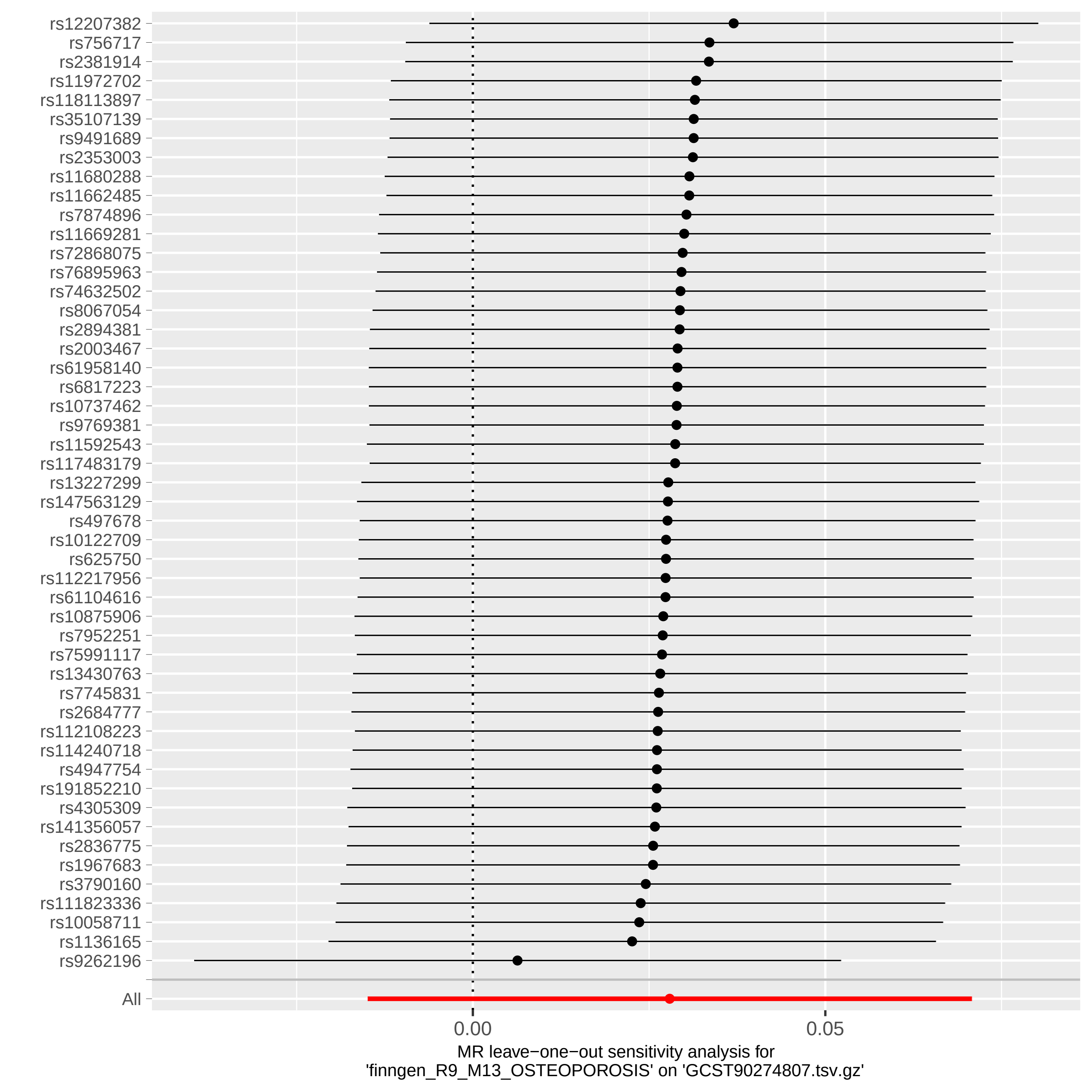

rs12207382
rs756717
rs2381914
rs11972702
rs118113897
rs35107139
rs9491689
rs2353003
rs11680288
rs11662485
rs7874896
rs11669281
rs72868075
rs76895963
rs74632502
rs8067054
rs2894381
rs2003467
rs61958140
rs6817223
rs10737462
rs9769381
rs11592543
rs117483179
rs13227299
rs147563129
rs497678
rs10122709
rs625750
rs112217956
rs61104616
rs10875906
rs7952251
rs75991117
rs13430763
rs7745831
rs2684777
rs112108223
rs114240718
rs4947754
rs191852210
rs4305309
rs141356057
rs2836775
rs1967683
rs3790160
rs111823336
rs10058711
rs1136165
rs9262196
All
0.00
0.05
MR leave−one−out sensitivity analysis for
'finngen_R9_M13_OSTEOPOROSIS' on 'GCST90274807.tsv.gz'
